# The Great Majority of Homologous Recombination Repair-Deficient Tumors Are Accounted for by Established Causes

**DOI:** 10.3389/fgene.2022.852159

**Published:** 2022-06-17

**Authors:** Paula Štancl, Nancy Hamel, Keith M. Sigel, William D. Foulkes, Rosa Karlić, Paz Polak

**Affiliations:** ^1^ Bioinformatics Group, Division of Molecular Biology, Department of Biology, Faculty of Science, University of Zagreb, Zagreb, Croatia; ^2^ Cancer Research Program, Research Institute of the McGill University Health Centre, Montreal, QC, Canada; ^3^ Icahn School of Medicine at Mount Sinai, New York, NY, United States; ^4^ Department of Human Genetics, McGill University Montreal, Montreal, QC, Canada; ^5^ Cancer Axis, Lady Davis Institute, Jewish General Hospital, Montreal, QC, Canada

**Keywords:** homologous recombination deficiency, HRDetect, CHORD, whole-genome sequencing, promoter methylation

## Abstract

**Background:** Gene-agnostic genomic biomarkers were recently developed to identify homologous recombination deficiency (HRD) tumors that are likely to respond to treatment with PARP inhibitors. Two machine-learning algorithms that predict HRD status, CHORD, and HRDetect, utilize various HRD-associated features extracted from whole-genome sequencing (WGS) data and show high sensitivity in detecting patients with *BRCA1/2* bi-allelic inactivation in all cancer types. When using only DNA mutation data for the detection of potential causes of HRD, both HRDetect and CHORD find that 30–40% of cases that have been classified as HRD are due to unknown causes. Here, we examined the impact of tumor-specific thresholds and measurement of promoter methylation of *BRCA1* and *RAD51C* on unexplained proportions of HRD cases across various tumor types.

**Methods:** We gathered published CHORD and HRDetect probability scores for 828 samples from breast, ovarian, and pancreatic cancer from previous studies, as well as evidence of their biallelic inactivation (by either DNA alterations or promoter methylation) in HR-related genes. ROC curve analysis evaluated the performance of each classifier in specific cancer. Tenfold nested cross-validation was used to find the optimal threshold values of HRDetect and CHORD for classifying HR-deficient samples within each cancer type.

**Results:** With the universal threshold, HRDetect has higher sensitivity in the detection of biallelic inactivation in *BRCA1/2* than CHORD and resulted in a higher proportion of unexplained cases. When promoter methylation was excluded, in ovarian carcinoma, the proportion of unexplained cases increased from 26.8 to 48.8% for HRDetect and from 14.7 to 41.2% for CHORD. A similar increase was observed in breast cancer. Applying cancer-type-specific thresholds led to similar sensitivity and specificity for both methods. The cancer-type-specific thresholds for HRDetect reduced the number of unexplained cases from 21 to 12.3% without reducing the 96% sensitivity to known events. For CHORD, unexplained cases were reduced from 10 to 9% while sensitivity increased from 85.3 to 93.9%.

**Conclusion:** These results suggest that WGS-based HRD classifiers should be adjusted for tumor types. When applied, only ∼10% of breast, ovarian, and pancreas cancer cases are not explained by known events in our dataset.

## Introduction

The recognition of biallelic germline or somatic mutations in *BRCA1/2* is, to date, one of the most clinically relevant and frequently used genetic biomarkers of homologous recombination repair deficiency (HRD) in the clinics (Dougherty et al., 2017; Hoppe et al., 2018). Patients harboring germline pathogenic variants (GPVs) in *BRCA1/2* have a higher risk of developing breast and/or ovarian cancer (Mersch et al., 2015). Patients with germline or somatic mutations have an enhanced benefit from targeted therapies such as platinum-based chemotherapy or poly (ADP-ribose) polymerase inhibitors (PARPi) (Hennessy et al., 2010). The terms “BRCAness” or “HRD phenotype” refer to tumors with similar clinicopathological and molecular characteristics to tumors with *BRCA1* and *BRCA2* GPVs (Lord and Ashworth, 2016). Gene alterations occurring in other homologous recombinant associated genes, such as *PALB2* (Tischkowitz et al., 2007; Thomas and Brown, 2015) and *RAD51C/D* (Kondrashova et al., 2017; Polak et al., 2017), have been linked to the HRD phenotype. Inactivation through promoter methylation of *BRCA1* and *RAD51C* has also been found to result in HRD tumors (Ruscito et al., 2014; Polak et al., 2017; Staaf et al., 2019), and these tumors also demonstrate increased sensitivity to PARPi and platinum (Kondrashova et al., 2018).

Advances in tumor sequencing resulted in the development of methods to identify HRD tumors independently of identifying the cause. Cancer genomes of patients with *BRCA1/2* mutations are enriched with particular mutational patterns as well as a high number of distinct LOH regions. In addition, *BRCA1/2-*deficient tumors include small deletions with ≥4 bp flanking homology. Several structural variations are typical of *BRCA1/2-*deficient cancer genomes, including deletions up to 100 kb, unclustered tandem duplications of ∼10 kb associated with *BRCA1* mutations (Willis et al., 2017), and deletions up to 1-10 kb in cancers are found in patients with *BRCA2* mutations (Degasperi et al., 2020). A specific single-base substitution signature (also known as single-nucleotide variants), referred to as COSMIC signature 3, is strongly associated with *BRCA1/2* deficiency (Polak et al., 2017).

Whole-genome sequencing (WGS) data enable the detection of different genomic alterations such as base substitutions, indels, rearrangements, and copy number aberrations, which are the result of homologous recombination deficiency. There are two HRD classifiers that are based on features extracted from WGS data. HRDetect (Davies et al., 2017) is a weighted logistic regression model based on six input features: the proportion of small deletions with microhomology at the breakpoint junction, HRD index based on genomic scars, COSMIC signatures 3 and 8, and two rearrangement signatures 3 and 5. This model was trained on *BRCA1/2*-null breast cancers. The classifier of Homologous Recombination Deficiency (CHORD) (Nguyen et al., 2020) is a random forest model that uses relative counts of somatic mutation contexts from WGS data.

Both classifiers classify >90% of tumors with biallelic inactivation via DNA mutation of *BRCA1/2* as HRD and have generally high accuracy as measured by AUC∼0.98 (area under the curve) (Davies et al., 2017; Nguyen et al., 2020). Mutations in *PALB2*, *RAD51C/D,* and *BARD1* are associated with HRD signatures (Polak et al., 2017; Matis et al., 2021) and account for a small fraction of non-*BRCA1/2-*mutated HRD cases (Golan et al., 2021). Nguyen et al. (2020), in the paper that introduced CHORD, reported that a substantial proportion (∼40%) of cancer samples identified as HR-deficient did not harbor any mutation in known HR-related genes (Nguyen et al., 2020), while Davies et al. (2017) reported more than 30% of these cases. These findings indicate that conventional testing for mutations in HR genes will miss a considerable number of HRD tumors where HRD is caused by unknown reasons.

The possible source of high unexplained cases could be either technical or biological. Both HRDetect and CHORD are continuous scores, designed to determine if a tumor exhibits HRD. Both use a universal threshold that was not optimized for specific cancer types. HRDetect threshold was developed based on the breast cancer dataset but this cut-off has been used for other cancer types. The CHORD study used a 0.5 cut-off. In addition, *BRCA1/RAD51C* promoter methylation is not measured in most WGS studies or on only one subset of these samples.

Here, we aim to examine the range of missing proportions of HRD samples across various three tumors where HRD is frequently reported (breast, ovarian and pancreas cancer) and determined the impact of cancer-type-specific thresholds as well as of promoter methylation *BRCA1/RAD51C* for an available subset of cases. To do so, we used published CHORD and HRDetect scores for these three cancers (Davies et al., 2017; Degasperi et al., 2020; Nguyen et al., 2020), as well as published HRDdetect scores for pancreas cancer (Golan et al., 2021) and CHORD scores that we calculated. In the case of ovarian and breast cancers, we limited our study to the subset of patients with available data for the methylation status of the *BRCA1/RAD51C* promoter. We determined the proportion of unexplained cases if we use cancer-type specific thresholds (for pancreas, ovarian, and breast cancer) and promoter methylation status (for ovarian and breast cancers).

## Materials and Methods

***Datasets*** *.* Studies that performed homologous-recombination deficiency detection analysis on the same samples using the CHORD (Nguyen et al., 2020) and HRDetect (Davies et al., 2017; Degasperi et al., 2020) classifiers were selected. From the selected studies, we made the largest unique intersection of sample names containing prediction scores of HR deficiency for both classifiers, CHORD and HRDetect. The dataset was divided into four major groups of HR-related cancers: breast, pancreatic, and ovarian (Supplementary Table S1), while other cancer types were put into a separate category (Supplementary Table S2) due to the low number of biallelic events and samples labeled as HRD. We included only breast and ovarian cancer samples that had verified *BRCA1*/2 with respect to methylation (Davies et al., 2017). The methylation status of HR-related gene promotors was considered to be an important underlying cause of HRD in tumors and we wanted to include only samples with validated methylation status to perform the downstream analysis. For the pancreatic dataset, we used 391 pancreatic samples whose data alongside the HRDetect classifier results were provided by Golan et al. (2021). For pancreatic samples, we ran the CHORD classifier using the default setting as it was previously described (Nguyen et al., 2020). The final combined dataset consisted of discrete datasets of 1) 371 breast cancers, 2) 66 ovarian cancers, 3) 391 pancreatic cancers, and 4) 1 238 samples belonging to other cancer types. For each sample in selected studies, we extracted the available methylation status of *BRCA1/2* genes for the breast and ovarian cancer samples alongside biallelic and monoallelic alternations in HR-related genes for all cancer types. We considered biallelic germline inactivation to be present when a germline pathogenic variant (GPV) was the first hit with the second hit being loss-of-heterozygosity (LOH) or somatic mutation. Somatic biallelic inactivation was considered where at least one hit was a somatic mutation, while promoter hypermethylation biallelic inactivation was defined as when one hit was promoter methylation and the other one was somatic or LOH. Monoallelic inactivation was considered when only one gene had any mutation other than LOH. Samples carrying biallelic inactivation in HR-related genes were considered to be true HR-deficient tumors. A detailed summary of all biallelic and monoallelic alterations in analyzed HR-related genes can be found in Supplementary Table S1 and Supplementary Table S2, alongside the source of information regarding these gene alterations.

***Assessment of the accuracy of CHORD and HRDetect classifiers through ROC and precision-recall curves***. To assess the accuracy of each classifier for each of the four major cancer types, we calculated receiver operating characteristics (ROCs) using the R function “roc” from package “pROC” (Robin et al., 2011) and precision-recall (PR) curves using R function “pr.curve” from package “PRROC” (Grau et al., 2015) by comparing CHORD and HRDetect probability scores against samples carrying biallelic inactivation in HR-related genes. Bootstrapping (2000 samples) was performed to estimate the 95% CI of the area under the ROC curve (AUC). Additionally, we compared the performance of these classifiers when no methylation data are available for breast and ovarian cancers to highlight the importance of promoter hypermethylation in HR-deficient tumors.

***Determining the optimal threshold.*** We applied a tenfold nested cross-validation approach to find the optimal threshold values of HRDetect and CHORD for classifying samples as HR-deficient or -proficient within breast, pancreatic, and ovarian cancers. The inner tenfolds were used to calculate the average optimal threshold, while the outer folds in the cross-validation process containing 10% of test data were used to assess the accuracy of the classification of HR-deficient samples. The reported optimal threshold for each classifier was calculated as the mean of all the average thresholds in outer loops for each cancer type.

***Statistical analysis.*** Probabilistic scores from CHORD and HRDetect classifiers were compared with Spearman correlation (Spearman, 1987) using R functions cor () or cor. test (). The one-sided partially overlapping samples z-test for dichotomous variables with R function “Prop.test” from package “Partiallyoverlapping” (Derrick, 2018) was used to determine the statistically significant differences in the proportion of samples classified as HRD samples with and without evidence between CHORD and HRDetect. An one-sided Fisher’s exact test using the R function “pairwise_fisher_test” from package “rstatix” (Kassambara, 2021) was used for testing the differences of explained and unexplained classifications between cancer types within each classifier. For comparison of the different ROC curves, we used the DeLong’s test (two-sided, paired-samples) for two correlated ROC curves using the R function “roc.test” from package “pROC” (Robin et al., 2011). Corrections for multiple hypothesis testing were done using Bonferroni correction, and adjusted *p*-values were reported. All the analyses were carried out in R statistical programming language version 4.1.0.

## Results

### Large Proportion of Homologous Recombination Repair Deficiency Classified Tumors Is Explained by Biallelic Inactivation of *BRCA1/2*

**FIGURE 1:**
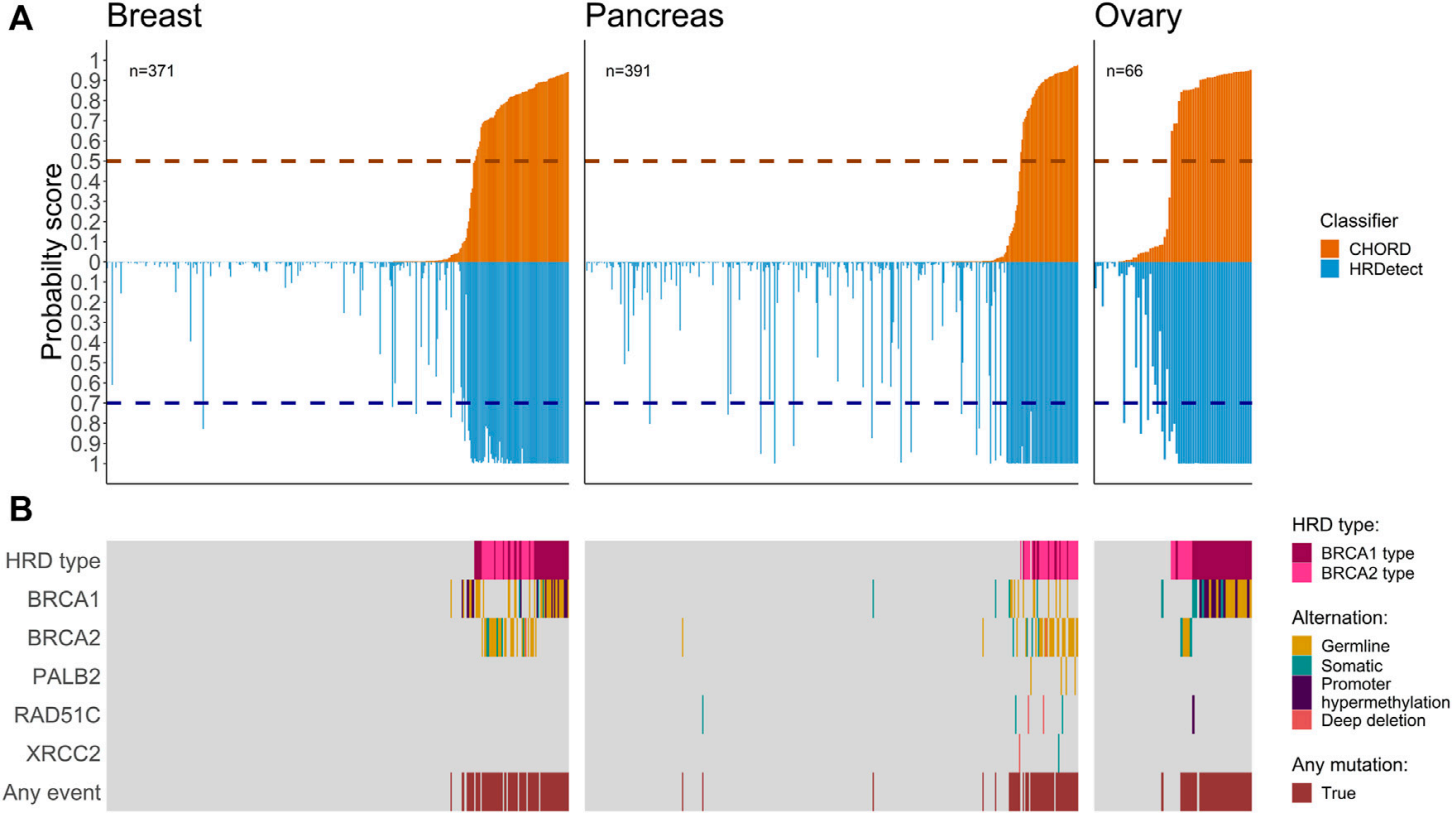
Co-mutation plots for breast, pancreas, and ovarian cancers. **(A)** Mirror bar plot showing the probability score of CHORD (orange) and HRDetect (blue) classifiers for each sample alongside the default threshold value for each classifier (horizontal dashed line, 0.5 for CHORD and 0.7 for HRDetect). Samples are ordered by the CHORD probability score from the lowest to the highest. **(B)** The biallelic inactivation in genes related to HR deficiency. HRD types (*BRCA1* and *BRCA2* types) were assigned by the CHORD classifier.

To investigate the performance of CHORD and HRDetect classifiers on the same samples, we utilized the classifiers’ results from previous studies (Davies et al., 2017; Degasperi et al., 2020; Nguyen et al., 2020; Golan et al., 2021) across 2,066 samples from 10 cancer types. Here, we have focused on comparing HRDetect and CHORD scores for a total of 828 tumors, composed of three cancers associated with HR deficiency: breast (*n* = 371), pancreatic (*n* = 391), ovarian (*n* = 66) (Figure 1A), while the remaining seven cancers are shown in the supplementary (Supplementary Figure S2, Supplementary Table S2). When comparing the probability score of a tumor possessing HRD for each sample, we see that CHORD and HRDetect have similar probability scores (Supplementary Figure S1, Spearman correlation of 0.67). Of the total 828 samples belonging to the three important HRD-related cancers, biallelic alterations (either somatic, germline, deep deletion, or promoter hypermethylation) of HR-related genes were found in 163 samples. As expected, samples with higher HRD probability scores (in both classifiers) had a higher number of biallelic inactivation events in *BRCA1/2* genes compared to samples with lower scores (Figure 1B). Somatic homozygous deletion, labeled as deep deletion, were observed in *BRCA2* in a single breast cancer patient and in pancreatic cancer (*RAD51B* (n = 2), *RAD51C* (*n* = 2) and *XRCC2* (*n* = 1)) (Supplementary Table S1).

Among other cancer types, we observed four prostate samples with high HRD scores from both classifiers containing biallelic inactivation in *BRCA1/2* and one biliary sample with a germline *BRCA1* alteration where both HRD scores were above default (Supplementary Figure S1). Due to lack of evidence for HR deficiency in other cancers and the smaller sample size of identified HRD samples, other cancers were excluded for the downstream analysis and we only benchmarked results for breast, ovarian, and pancreatic cancer samples.

We proceeded to compare the fraction of HRD classified cases that are explained by the different types of biallelic inactivation in *BRCA1/2* based on HRDetected and CHORD. The most abundant biallelic inactivation patterns in the dataset included g*BRCA1/2* (n = 52 + 54) and s*BRCA1/2* mutations (*n* = 12 + 11) (Supplementary Table S1). The *BRCA1* promoter methylation status was available only for breast and ovarian (*n* = 23) and it accounted for a significant number of the total biallelic events (23 out of 175, 12.8% (95% CI [8.7–19.3])). Nearly all of the cases with known biallelic inactivation (157 out of 163, 96.4% (95% CI [91.8–98.5])) were in tumors that are above the default threshold of either of the classifiers.

**FIGURE 2:**
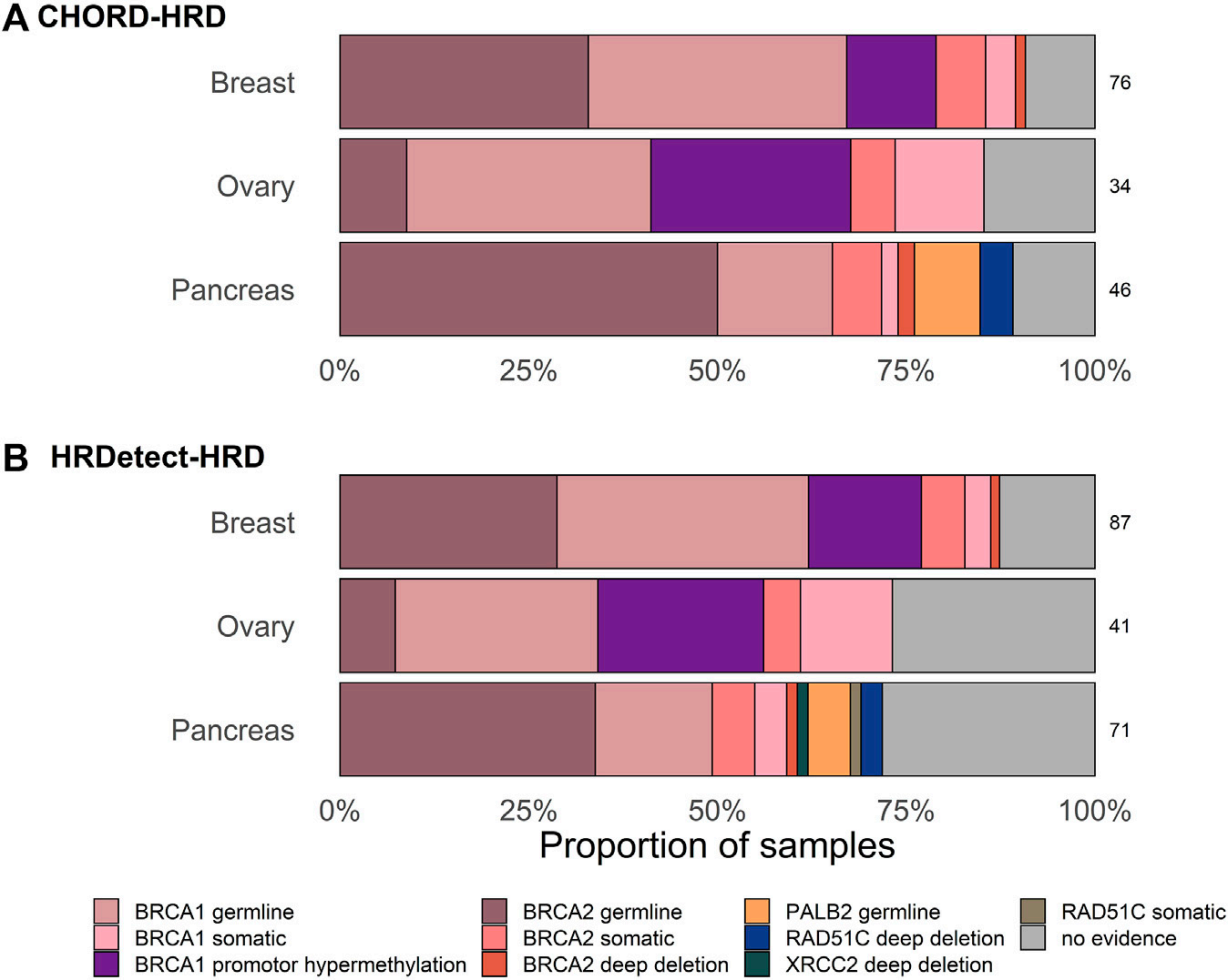
Proportion of samples with and without biallelic alteration in HR-genes classified as HR-deficient with default threshold of **(A)** HRDetect of 0.7 and **(B)** CHORD classifiers of 0.5. Only one alteration in the gene is shown per sample based on the hierarchical order of genes as follows: *BRCA1*, *BRCA2*, *RAD51C*, *PALB2,* and *XRCC2*.

The largest proportion of unexplained HRD cases was observed in ovarian cancer (14.7%, 95% CI [5.5–31.8]) using CHORD and in pancreatic samples (28.2%, 95% CI [59.7–81.6]) using HRDetect (Table 1; Figure 2). Larger fractions of unexplained cases were obtained using HRDetect compared to CHORD with the default threshold value (one-sided *z*-test for partially overlapping samples, *p*-value < 10^–13^) (Figure 2), ranging from around 10 to 28% depending on the cancer type. When looking at each classifier closely, we see that the highest difference is between breast and pancreatic cancers and HRD unexplained cases for HRDetect (one-sided Fisher’s exact test, *p*-value = 0.0375). Multiple biallelic inactivation events can occur in HR genes in the same patients; for instance, one ovarian sample contained *sBRCA1* and promoter hypermethylation of *RAD51C*, while a pancreatic sample had a somatic deep deletion of both *RAD51B* and *RAD51C* (Supplementary Table S1).

**TABLE 1:** Summary of tumor samples classified to possess HRD by CHORD and HRDetect within an individual cancer type.

	Evidence of Biallelic Inactivation		No Evidence of Bi-allelic Inactivation	
	Count	Proportion (95% CI)	Count	Proportion (95% CI)
**CHORD**				
Breast	69	90.8 (81.4-95.9)	7	9.2 (4.1-18.6)
Ovary	29	85.3 (68.2-94.5)	5	14.7 (5.5-31.8)
Pancreas	41	89.1 (75.6-95.9)	5	10.9 (4.1-24.4)
**HRDetect**				
Breast	76	87.4 (78.1-93.2)	11	12.6 (6.8-21.9)
Ovary	30	73.2 (56.8-85.2)	11	26.8 (14.8-43.2)
Pancreas	51	71.8 (59.7-81.6)	20	28.2 (18.4-40.3)

### Performance of CHORD and HRDetect Classifiers

**FIGURE 3:**
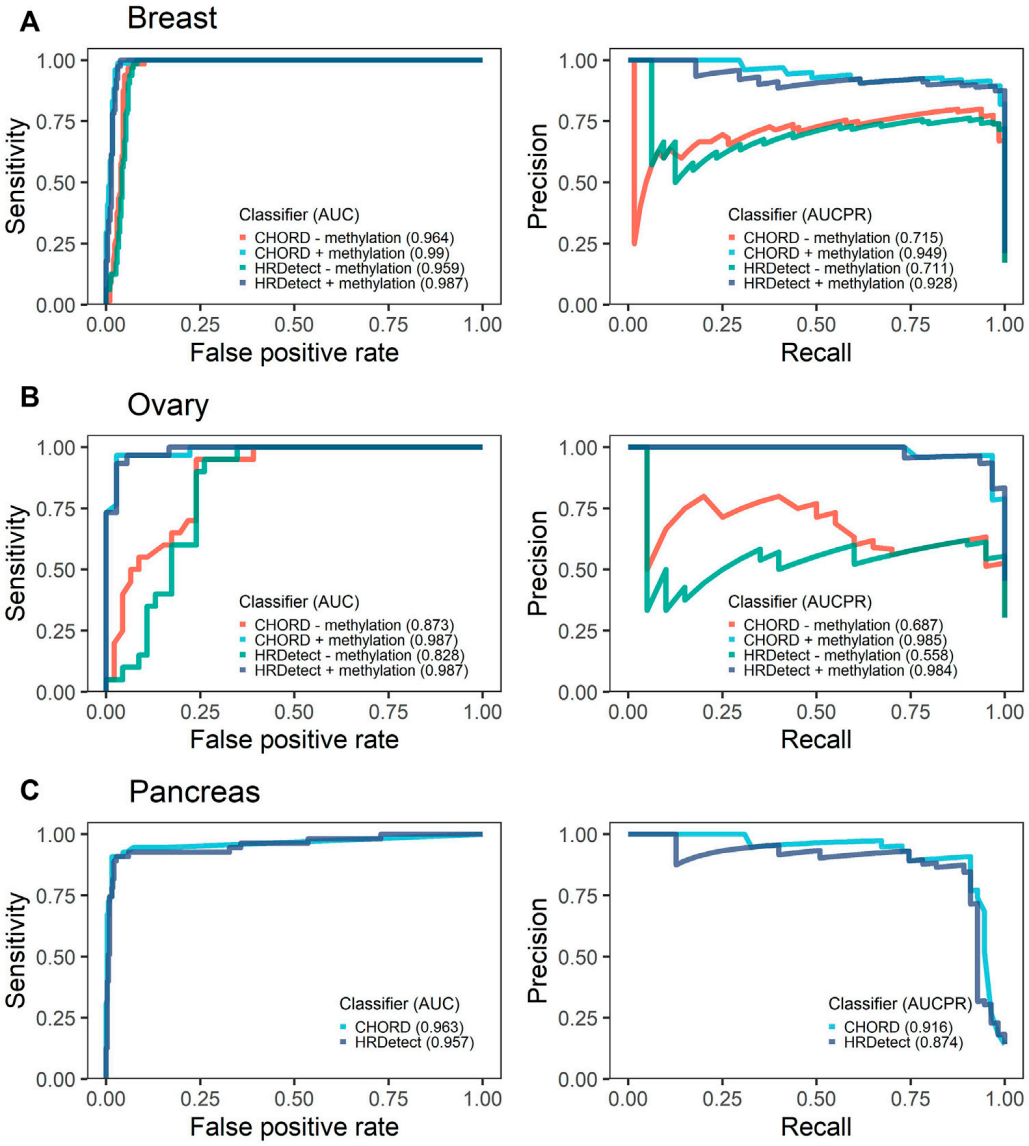
Receiver operating characteristics (ROCs) with the respective area under the curve (AUC) and precision-recall curves (PR) with the area under the precision-recall curve (AUCPR) showing the performance of CHORD and HRDetect classifier with and without methylation data for breast **(A)** and ovarian cancers **(B)**. Pancreas **(C)** cancer data do not have methylation data.

As previously reported, both classifiers, CHORD and HRDetect, achieved exceptional performance in identifying biallelic events in breast and ovarian cancer types as shown by the high area under the ROC curve (AUC) above 0.96 and 0.9, respectively (Figure 3). In addition, we calculated the area under the precision-recall curve (AUPRC) that was high and well above 90% across all cancer types. No statistically significant difference was detected between CHORD and HRDetect AUC values (*p* > 0.05, DeLong’s test).

### Impact of Exclusion of Promoter Methylation on the Performance

To assess the importance of promoter methylation in the evaluation of HRD classifiers' performance, we removed the methylation data of *BRCA1/RAD51C* promoters in breast and ovarian the only cancer types for which methylation data were available. We observed a significant drop in classifiers’ performance for breast and ovarian samples (Figure 3). In ovarian cancer, the drop in AUC values was significantly affected, falling from 0.987 to 0.873 for CHORD (*p*-value = 0.044, DeLong’s test) and from 0.987 to 0.828 for HRDetect (*p*-value = 0.011, DeLong’s test). In contrast, the breast cancer AUC values were still above 0.96 for both classifiers (*p*-value = 0.057 for CHORD and 0.055 for HRDetect, DeLong’s test) and AUPRC values were slightly above 0.7 compared to 0.949 when methylation status was included.

### Revisiting Threshold Values for Homologous Recombination Repair Deficiency Classification of Different Cancer Types

The current threshold of HRDetect( 0.7) was determined based on the breast dataset, while CHORD 0.5 was arbitrarily chosen. Considering different machine-learning algorithms underlying CHORD and HRDetect for classifying HRD in samples and different training data, we sought to determine an optimal threshold value for the individual cancer types in our cohort. For each cancer type and classifier, we performed 10-fold nested cross-validation to calculate the optimal threshold value (given in detail in the Methods section). The accuracy of both classifiers with default threshold values was similar across cancers, while the most considerable increase was detected in ovarian cancer (accuracy CHORD 0.91 and HRDetect 0.83) (Table 2). Cancer-type-specific (optimal) threshold values differ from the classifiers’ default ones, but the overall accuracy improves slightly or remains the same. The only exception is the optimal value of HRDetect in ovarian cancer where the accuracy improved by 12%. The number of samples with evidence of bi-allelic alterations in HR-related genes and classification as HR deficient by the classifiers were more abundant in optimal values of the CHORD classifier in breast and pancreatic cancers compared to the default threshold in the same cancer type. The proportion of classified HRD cases in the dataset without known biallelic evidence decreased for both CHORD, 10.9–8.9%, and HRDetect, 21.1–12.3%. Monoallelic mutations were found in pancreatic cancer (Supplementary Figure S3). Using default threshold values, the majority of monoallelic mutation in HR-related genes occurs in homologous recombination proficient (HRP) samples, where HRDetect has more HRD unexplained cases and two monoallelic mutations in HR-related genes. The monoallelic alterations were detected in HRD-labeled samples only with HRDetect with default and an optimal threshold value.

**TABLE 2:** Summary table of confusion matrix results with accuracy for default and optimal (cancer-type-specific) threshold values of CHORD and HRDetect classifiers for classifying samples as homologous recombination deficient (HRD) or homologous recombination proficient (HRP).

	CHORD						HRDetect					
	HRD		HRP				HRD		HRP			
**Bi-allelic Evidence**	**Yes**	**No**	**Yes**	**No**	**Threshold**	**Accuracy**	**Yes**	**No**	**Yes**	**No**	**Threshold**	**Accuracy**
**Breast**												
default	69	7	9	286	0.50	0.96	76	11	2	282	0.70	0.96
optimal	75	9	3	284	0.10	0.97	77	11	1	282	0.68	0.97
**Ovary**												
default	29	5	1	31	0.50	0.91	30	11	0	25	0.70	0.83
optimal	29	1	1	35	0.84	0.97	29	2	1	34	0.99	0.95
**Pancreas**												
default	41	5	14	331	0.50	0.95	51	20	4	316	0.70	0.94
optimal	49	5	6	331	0.13	0.97	50	9	5	327	0.98	0.96

a HRD and HRP categories are determined by CHORD and HRDetect based on default or optimal (cancer-type-specific) thresholds.

## Discussion

Our study shows an integrated overview of detecting homologous recombination deficiency in cancers using CHORD and HRDetect classifiers. Here, we have mainly focused on three cancers most commonly associated with HRD: breast, ovarian, and pancreatic cancers. We observed that biallelic inactivation of genes explains a large fraction of samples possessing HRD when using a universal default threshold, as was demonstrated in previous studies (Davies et al., 2017; Nguyen et al., 2020; Golan et al., 2021). However, around 10–28% of patients without known underlying causes were detected by these classifiers despite their high performance based on the default threshold. In this study, we found that by applying a cancer-type-specific threshold the number of unexplained cases reduced to around 8.9–12.3% without decreasing the sensitivity of 96%. We estimate that in this dataset up to ∼10% of HRD cases are caused by types of alterations that still have not been associated with HRD and therefore gene-centric testing for mutations in HR genes will likely fail to identify them. Similar results apply to the analysis of other cancer types in which the HRD cancers are rarer in comparison to the four well-known HRD cancers. The low number of HRD mutations in prostate samples and other cohorts did not allow the determination of a reliable cancer-type-specific threshold. The small fraction of unexplained cases is consistent with our previous proposal (Foulkes and Polak, 2019; Matis et al., 2021) that if alterations in novel genes lead to HRD, taken together, they will all account for only a very small proportion of all HRD cases.

The different cut-offs that we observed may be due to subtle differences across cancer in the mutational landscape even for tumors with different same gene defects, especially in mutational signatures (Degasperi et al., 2020). Furthermore, as it was highlighted by Nguyen et al. (2020), additional threshold optimization and validations are also required when applying classifiers to WGS data generated by other variant calling pipelines. Our cohort contained data generated by various pipelines for CHORD and HRDetect in each cancer type, which may affect the overall comparison of results between these classifiers. In addition to the threshold value, it is important to investigate other features affecting the mutation landscape of tumors, such as deficiency in mismatch repair (MMR), which may have a negative impact on the overall performance of classifiers in specific tumors. It was noted by Golan et al. (2021) that one pancreatic sample with biallelic inactivation in *BRCA2* and *PMS2* (responsible for MMR) was misclassified by HRDetect and CHORD classifier and had both scores near zero.

In addition to cancer-type-specific thresholds that reduce the number of unexplained cases, we demonstrated the importance of including the promoter methylation status of *BRCA1* and *RAD51C* in order to evaluate the fraction of HRD cases that are explained by known causes. In breast and ovarian cancers, for which methylation analysis is most often conducted, promoter methylation of *BRCA1* accounts for at least 20% of explained biallelic inactivation cases of HRD, labeled by either of the classifiers, and lack of methylation data significantly affects the performance of classifiers. The proportion of unexplained cases in other cancer types may have been reduced if methylation analysis data existed, especially in pancreatic cancer where some detected monoallelic PVs could have other events such as promoter methylation that would explain their HRD. These observations highlight the advantage of using these classifiers alongside conventional testing for patient selection and stratification in clinics, as was already suggested by several studies (Zhao et al., 2017; Staaf et al., 2019; Chopra et al., 2020). The relationship between the presence of HRD and response to therapies such as PARP inhibitors is not precise and there is currently no “ground truth” for measuring HRD. Resistance to PARP inhibitors can co-exist with HRD (Dias et al., 2021), so the presence of HRD is not by itself a direct predictor of response to PARP inhibitors and other drugs such as platinum that cause double-strand DNA breaks. Combinations of different approaches such as WGS-based, FDA-approved assays, and newer functional assays such as the RAD51 foci assay (Pellegrino et al., 2022) will ultimately lead to a better selection of HRD patients for appropriate therapies. Hence, our review re-analysis emphasizes the power of both CHORD and HRDetect in the stratification of patients possessing HRD phenotype across various cancers, as well as the importance of identification and further validation of new unrevealed oncogenic mutations.

## Data Availability

The original contributions presented in the study are included in the article/Supplementary Material; further inquiries can be directed to the corresponding author.
