# Supplementary material for: The Great Majority of Homologous Recombination Repair-Deficient Tumors Are Accounted for by Established Causes
Source: Front Genet. 2022 Jun 17;13:852159. doi: 10.3389/fgene.2022.852159 (PMC9247292; doi:10.3389/fgene.2022.852159)
Supplement: Supplementary file 1 [file DataSheet1.PDF]

Supplementary Material

1 Supplementary Figures

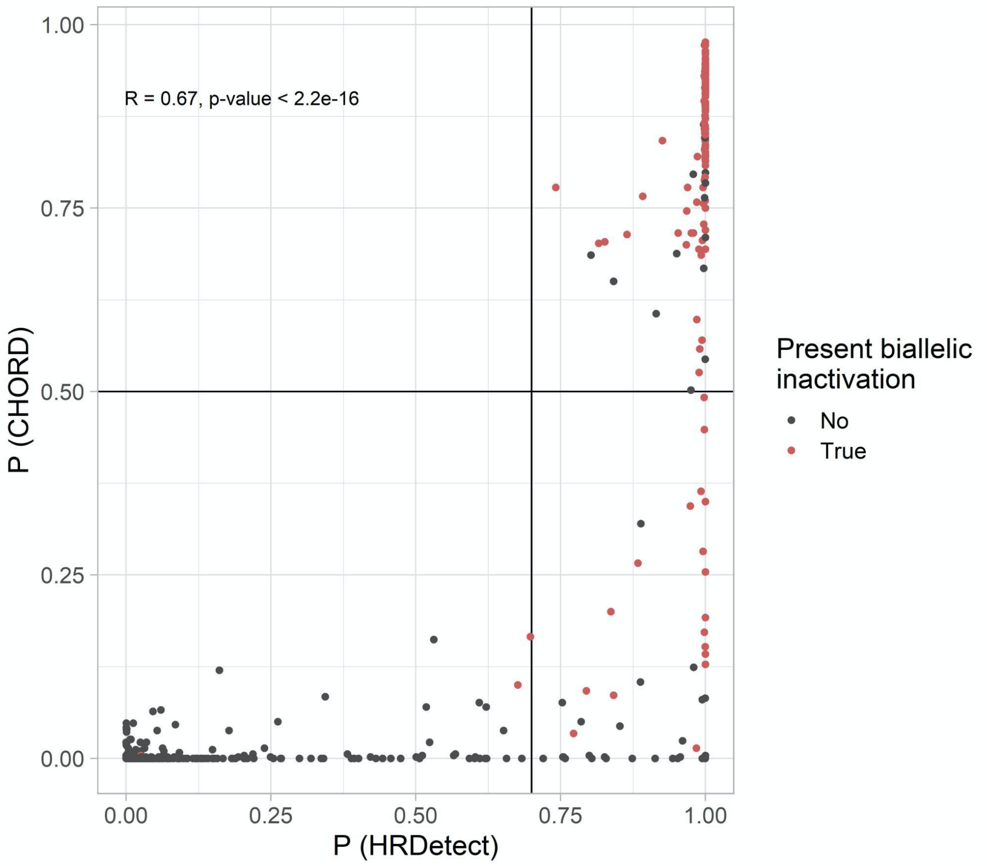

Supplementary Figure 1. Scatterplot for CHORD and HRDetect probability scores for breast, ovarian and pancreatic cancer data with presence of any biallelic inactivation in *BRCA1/2*, *PALB2*, *RAD51C* and *XRCC2*. Spearman correlation is 0.67, p-value < 2.2e-16

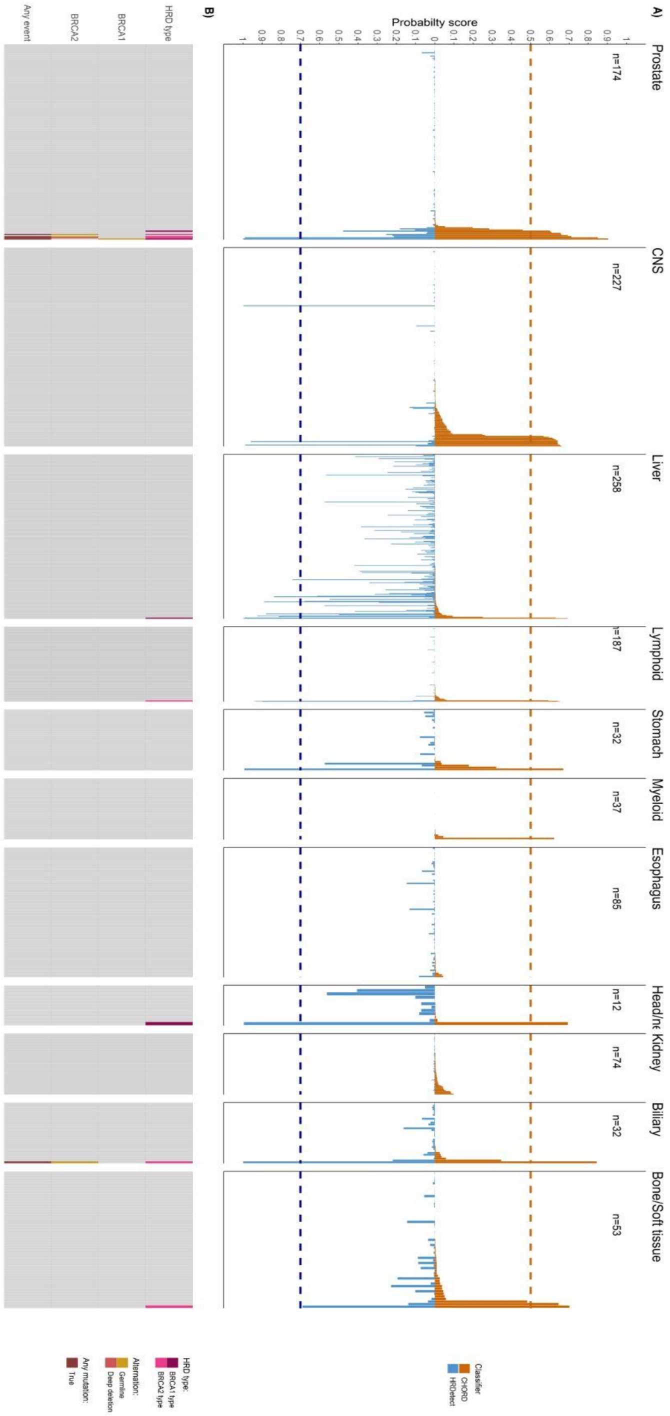

Supplementary Figure 2. Co-mutation plots for other cancers. A) Mirror bar plot showing the probability score of CHORD (orange) and HRDetect (blue) classifiers for each sample alongside default threshold value for each classifier (horizontal dashed line, 0.5 for CHORD and 0.7 for HRDetect). B) The biallelic inactivation in genes related to HR deficiency. HRD types (*BRCA1*-type and *BRCA2*-type) were assigned by CHORD classifier. C) The monoallelic inactivation in samples which do not have biallelic inactivation of any gene as evidence for HR deficiency.

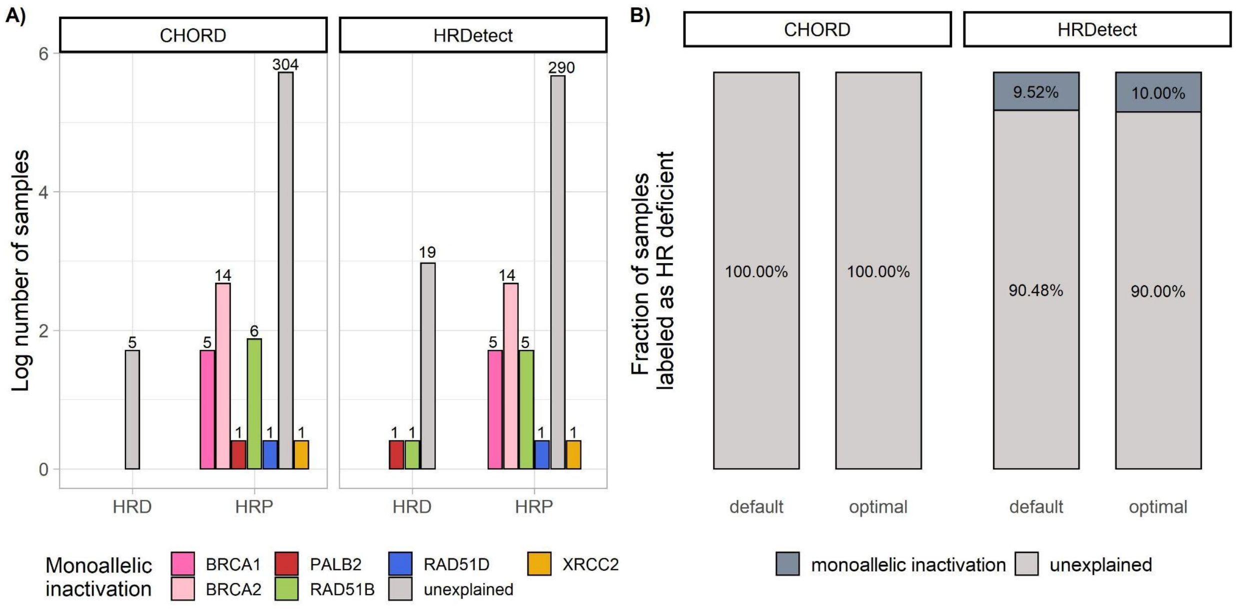

Supplementary Figure 3. Monoallelic mutations (green) in genes in pancreatic cancer patients without biallelic inactivation in HR-related genes labeled as HR deficient (HRD) and HR proficient (HRP) using default and optimal threshold values of CHORD and HRDetect classifier
